# Transcriptomic regulation of pancreatic acinar cell homeostasis and plasticity

**DOI:** 10.1042/BST20253124

**Published:** 2026-06-04

**Authors:** Xin Zhang, Christopher Luo, Kevin Coughlin, Feng-Qian Li, Ken-Ichi Takemaru, Ledong Wan

**Affiliations:** 1 Department of Pharmacological Sciences, State University of New York at Stony Brook, Stony Brook, NY 11794, U.S.A.; 2 Stony Brook Cancer Center, Renaissance School of Medicine at Stony Brook University, Stony Brook, NY 11794, U.S.A.; 3 College of Arts & Sciences, Cornell University, Ithaca, NY, U.S.A.

**Keywords:** Acinar Cell Homeostasis, Acinar-to-Ductal Metaplasia, Pancreas, Plasticity, Transcriptomic Regulation

## Abstract

Pancreatic acinar cells are highly specialized secretory epithelial cells in which coordinated transcriptional, epigenetic, and post-transcriptional regulatory mechanisms maintain digestive enzyme production, polarized architecture, and lineage fidelity. This homeostatic network preserves acinar identity while enabling rapid adaptation to physiological stress. Disruption of these regulatory mechanisms triggers acinar-to-ductal metaplasia (ADM), a reversible reprogramming state that supports acinar cell survival and regeneration after acute injury. However, if ADM persists under chronic inflammation or oncogenic KRAS activation, it can facilitate the initiation of pancreatic ductal adenocarcinoma. Elucidating these mechanisms offers opportunities to restore acinar cell homeostasis, reverse ADM, and prevent neoplastic transformation. In this review, we summarize current knowledge on the transcriptional, epigenetic, and post-transcriptional regulation of acinar cell homeostasis and plasticity, with emphasis on their roles in ADM.

## Introduction

**Figure 1:**
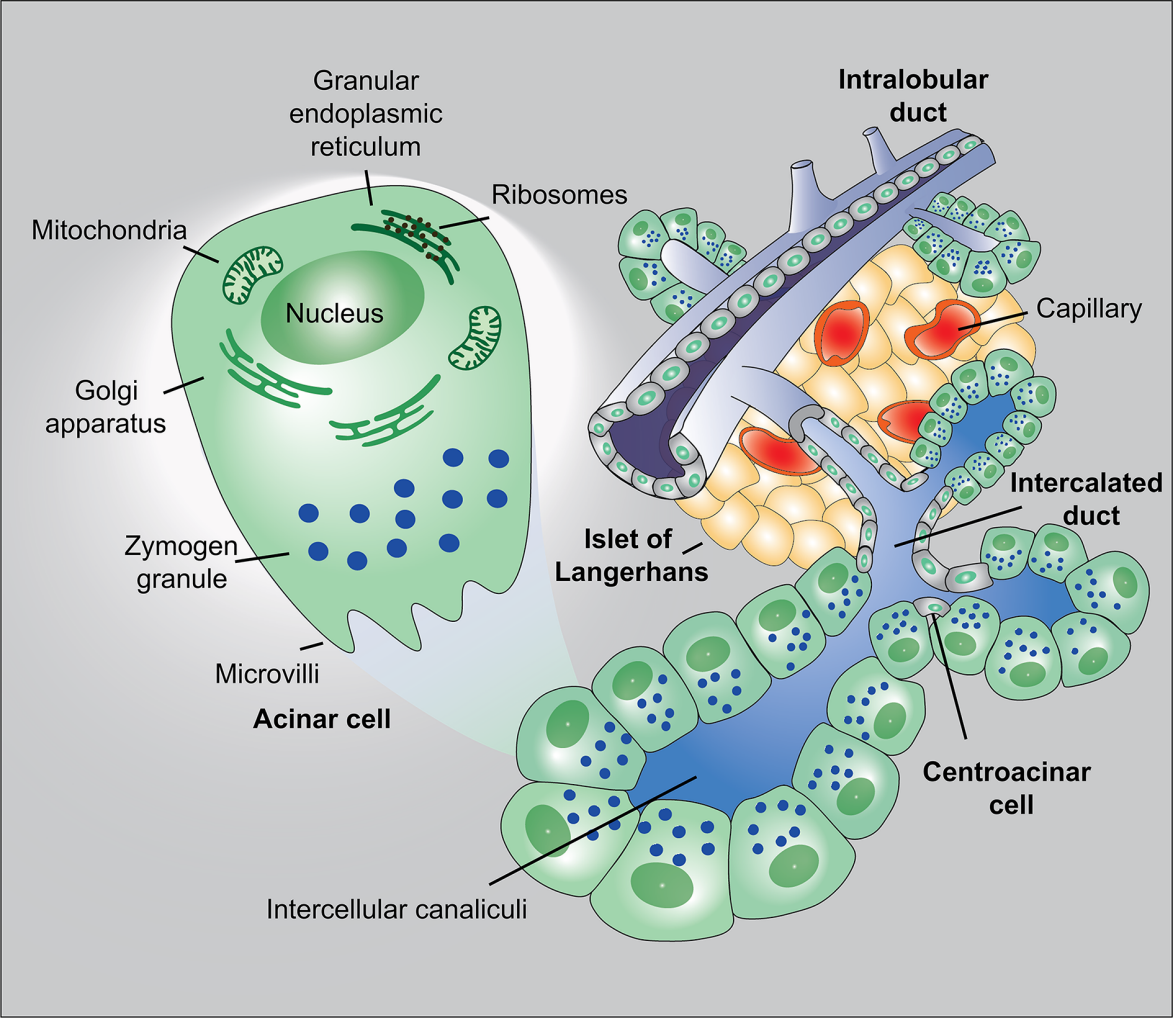
Organization of the pancreas. The pancreas contains an endocrine compartment, composed of the islets of Langerhans that contain multiple hormone-producing cell types (including insulin-secreting β-cells), and an exocrine compartment consisting of acinar cells and a branching ductal network. Acinar cells are organized into spherical acini surrounding a central lumen and secrete digestive enzymes (amylase, lipase, and proteases) into intercalated ducts. These ducts converge into intralobular and interlobular ducts, ultimately joining the main pancreatic duct.

Pancreatic acinar cells are highly differentiated epithelial cells that comprise over 90% of the total cells within the pancreas. They are responsible for the synthesis, packaging, and intracellular trafficking of digestive enzymes, which are stored in zymogen granules and secreted into the small intestine to facilitate nutrient digestion (Figure 1). These include amylase for carbohydrate hydrolysis, lipase for lipid breakdown, and a broad spectrum of proteases such as trypsinogen, chymotrypsinogen, and elastase [1–5]. Beyond their digestive functions, acinar cells demonstrate remarkable plasticity, enabling them to undergo acinar-to-ductal metaplasia (ADM) in response to injury, inflammation, or oncogenic stress [6,7]. ADM promotes acinar cell survival and regeneration after acute injury, but when sustained by chronic inflammation or oncogenic KRAS activation, it can serve as a critical precursor to pancreatic intraepithelial neoplasia (PanIN) and, ultimately, pancreatic ductal adenocarcinoma (PDAC) [3,7–11].

Given their central role in both normal physiology and disease initiation, acinar cells represent a critical hub for pancreatic homeostasis and pathogenesis. Therefore, elucidating the transcriptome regulatory networks that maintain acinar identity, and how their disruption drives pathological transformation, will not only deepen our understanding of pancreatitis and PDAC, but also lay the groundwork for therapeutic interventions to re-establish homeostasis, reverse ADM, and prevent neoplastic transformation.

## Pancreatic development and acinar lineage specification

During mouse embryogenesis, definitive endoderm (DE) is generated from epiblast cells that ingress through the primitive streak during gastrulation (approximately E6.5–E7.5), a process characterized by dynamic widespread intercalation of epiblast-derived DE cells with visceral endoderm cells [12]. As anterior–posterior patterning of the gut tube is established, pancreatic specification is initiated around E8.5 with the onset of pancreatic and duodenal homeobox 1 (*Pdx1*) expression in the posterior foregut endoderm [13]. This marks the beginning of a coordinated developmental program that gives rise to exocrine, endocrine, and ductal cell types [14]. PDX1-positive pancreatic epithelial cells subsequently undergo expansion and early morphogenetic organization, contributing to the progressive establishment of the pancreatic endoderm (PE) by approximately E9.5–E10.5 [15].

Pancreatic development subsequently proceeds through two major transitional phases, termed the primary and secondary transitions. During the primary transition (E9.5–E12.5), pancreatic progenitor cells expand while maintaining multipotency and initiating early lineage specification [16–18]. At early stages (E9.5–E10.5), *Pdx1^+^ Ptf1a^+^* cells within the emerging pancreatic buds represent multipotent progenitor cells (MPCs) that give rise to all major pancreatic lineages, including exocrine, endocrine, and ductal cells *in vivo* [16,19]. The secondary transition (E13.5–E15.5) constitutes a major wave of differentiation, during which the branching epithelium becomes compartmentalized into tip and trunk domains [16,18,20]. At this stage, a distal tip progenitor population defined by *Pdx1^+^ Ptf1a^+^ Cpa1^+^* expression gives rise to all major pancreatic lineages [16,21]. Progressive tissue outgrowth and differentiation subsequently establish the trunk epithelium. This tip–trunk segregation forms the foundation for lineage specification: cells within the distal tip domain preferentially adopt the acinar fate, marked by sustained *Ptf1a* expression, whereas trunk cells give rise to ductal and endocrine lineages [16,21].

Commitment to the acinar lineage is driven by a PTF1-centered transcriptional network, with PTF1 complex assembly during differentiation enabling mature acinar secretory gene expression [22–24]. GATA4, together with GATA6, further supports progenitor expansion, branching morphogenesis, and acinar differentiation [25,26]. As differentiation proceeds, acinar cells undergo structural maturation characterized by expansion of rough endoplasmic reticulum (RER), establishment of apical–basal polarity, and accumulation of zymogen granules [27,28] These regulatory networks promote acinar cell differentiation and maturation, establishing a specialized secretory identity.

## Homeostasis of the acinar cell transcriptome

**Figure 2:**
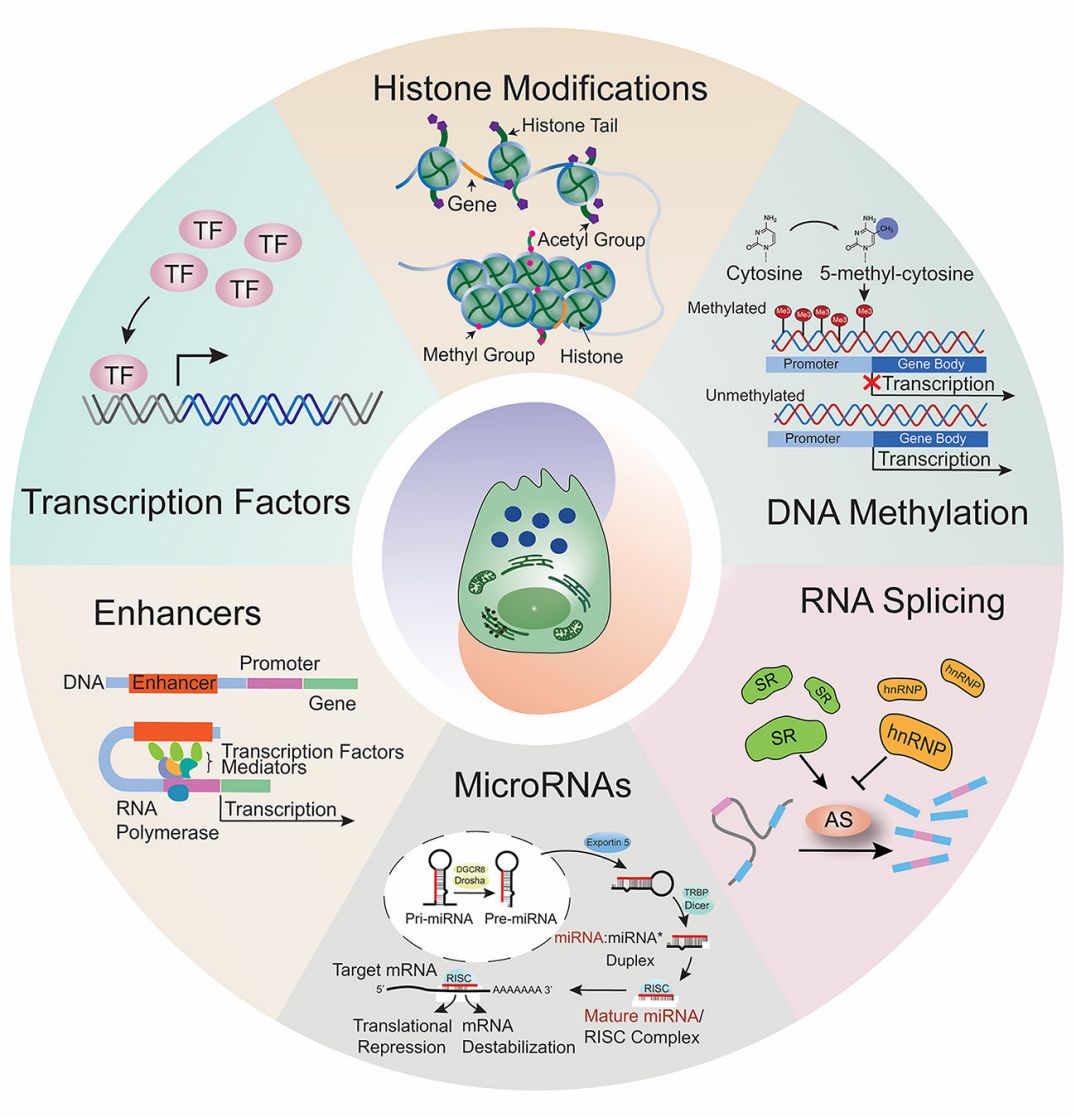
Acinar cell transcriptome homeostasis is coordinated by multiple regulatory mechanisms. Acinar cells preserve their highly specialized secretory identity while balancing the demands of digestive enzyme production with the need to adapt rapidly to physiological fluctuations and stress. This balance is maintained by an integrated network of transcriptional, epigenetic, and post-transcriptional programs that safeguard lineage fidelity while permitting controlled plasticity for tissue repair.

Acinar cell transcriptome homeostasis is sustained by integrated transcriptional, epigenetic, and post-transcriptional regulation. Lineage-defining transcription factors establish interconnected networks that maintain acinar identity and suppress cell dedifferentiation [29,30]. These programs are reinforced by permissive chromatin landscapes at acinar-specific loci [24,31,32]. At the post-transcriptional level, mechanisms such as alternative splicing, translational control, and microRNA (miRNA)-mediated regulation fine-tune gene expression to support the high secretory demands of acinar cells while safeguarding cell identity (Figure 2) [33–35].

## Transcriptional regulation

### Transcription factors

#### PTF1A

Pancreas transcription factor 1 subunit alpha (PTF1A) functions early in both endocrine and exocrine pancreas development, and later becomes essential for proper exocrine differentiation and the maintenance of acinar cell fate [19,36–38]. Its activity is regulated in a cell type- and stage-dependent manner. PTF1A is critical for the primary transition of early pancreatic MPCs and the later secondary transition, particularly within pro-acinar progenitors located in the tip epithelium [16,19,38]. From E12.5 onward, PTF1A increasingly promotes acinar cell specification and becomes progressively restricted to the acinar lineage, resulting in its exclusive expression in acinar cells in the differentiated pancreas [38,39].

As a basic helix–loop–helix (bHLH) transcription factor, PTF1A assembles into a trimeric PTF1 complex with E-proteins (E12 or E47) and either RBPJ or its pancreas-restricted paralog RBPJL [40]. During early pancreatic development, PTF1A binds to RBPJ to activate the expression of acinar lineage genes, including *Rbpjl*. As development progresses, RBPJL replaces RBPJ to form the PTF1A/RBPJL complex, which shifts the complex’s binding specificity to promoters of mature acinar-specific genes, such as those encoding secretory digestive enzymes [24,40]. The importance of this regulatory mechanism is underscored by studies in mice expressing the PTF1A^W298A^ mutant, which disrupts PTF1A binding to RBPJ but not RBPJL, resulting in absence of the pancreatic ventral bud and delayed growth of the dorsal bud, phenocopying PTF1A-null mutants [23]. These findings establish PTF1A as a central transcriptional regulator governing acinar lineage specification and differentiation.

#### GATA4 and GATA6

In vertebrates, members of the GATA (guanine–adenine–thymine–adenine) zinc finger transcription factor family recognize the consensus binding site A/T-GATA-A/G within the promoter regions of numerous target genes [41]. Among them, GATA4 and GATA6 play critical roles in pancreatic development and acinar differentiation. They directly activate the transcription of acinar-specific genes, including those encoding digestive enzymes and components of the secretory machinery [29,42]. During early embryogenesis, both factors are expressed in the foregut endoderm and early pancreatic epithelium and are critical for the formation of the pancreatic buds [43]. While deletion of either *Gata4* or *Gata6* alone in mice does not significantly impair pancreatic development, concurrent loss of both genes results in pancreatic agenesis, demonstrating their essential and functionally redundant roles in pancreas specification [25].

Despite partial functional redundancy, GATA4 and GATA6 make unequal contributions to pancreatic development, as demonstrated by dosage-dependent genetic studies. Double *Gata4/Gata6* mutant mice failed to develop a pancreas, died shortly after birth, and exhibited hyperglycemia [26]. Notably, deletion of a single *Gata6* allele in *Gata4* conditional knockout mice markedly reduces pancreatic mass with severe acinar loss, whereas one *Gata4* allele in *Gata6* knockout mice permits normal development, indicating their different functional contributions. Moreover, GATA4 expression is preferentially enriched in the tip epithelium, where acinar progenitors are localized, whereas GATA6 exhibits broader expression throughout the pancreatic epithelium [42].

In the adult pancreas, GATA6 continues to support acinar cell identity by regulating transcriptional networks involving RBPJL and MIST1 [44]. Loss of GATA6 increases their susceptibility to oncogenic KRAS^G12V^, thereby promoting ADM and accelerating tumor initiation and progression [45]. GATA6 exerts its tumor-suppressive function by maintaining acinar differentiation, restraining pro-inflammatory signaling, and directly repressing oncogenic transcriptional programs.

#### MIST1

MIST1 (also known as BHLHA15) is a bHLH transcription factor that plays a critical role in acinar maturation, promoting the expansion of the secretory apparatus and the proper organization of zymogen granules [27]. Unlike canonical bHLH proteins, MIST1 contains an N-terminal repressor domain that allows it to antagonize other bHLH transcription factors by forming inactive heterodimers [46]. MIST1 expression is observed in a broad range of secretory tissues, including pancreatic acinar cells, serous or seromucous cells of the salivary glands, chief cells of the stomach, and secretory cells of the prostate and seminal vesicles [47]. *Mist1*-deficient acinar cells display loss of polarity, disorganized granules, and increased susceptibility to stress [48]. In addition, *Mist1*-deficient mice show dramatic transcriptional upregulation of several genes associated with pancreatic injury and pancreatitis, including *Reg1*/*PSP*, *PAP1*/*RegIII*, and *p8* [46,49–51]. In contrast, ectopic MIST1 expression inhibits cellular proliferation through induction of p21, a cyclin-dependent kinase inhibitor encoded by the *Cdkn1a* gene [52]. Target gene analysis confirmed that MIST1 regulates *Cdkn1a* gene expression, and the activation is dependent on specific transcription factor Sp1-binding sites located within the *Cdkn1a* proximal promoter. Analysis of intact pancreata also revealed that acinar cells of *Mist1*-KO mouse exhibit a higher proliferative index compared with control littermates [52].

#### PDX1

PDX1 (also known as IPF1) is a master transcription factor essential for pancreas organogenesis, exocrine function, and endocrine lineage specification [13,53,54]. During embryonic development, PDX1 marks multipotent pancreatic progenitors and drives the formation of all three pancreatic lineages including acinar, ductal, and endocrine cells [15,55]. In the differentiated pancreatic cells, PDX1 expression is largely restricted to β-cells, where it regulates insulin transcription and glucose responsiveness [56] Low levels of PDX1 are also maintained in acinar cells to support cellular identity, digestive enzyme expression, and stress adaptation. Both upregulation and loss of PDX1 disrupt acinar homeostasis, compromising lineage identity and promoting ADM [57,58].

### Epigenetic landscape

#### Chromatin accessibility and enhancer usage

Chromatin accessibility is a fundamental determinant of gene regulation. It reflects the extent to which DNA is exposed and available for transcription factor and regulatory protein binding, a state largely regulated by nucleosome positioning and post-translational histone modifications [59]. Open chromatin regions are typically associated with active regulatory elements, such as enhancers—non-coding DNA sequences that increase the transcription of target genes, often across long genomic distances [60,61]. Enhancers function by recruiting transcription factors and coactivators, facilitating the formation of chromatin loops that establishes physical proximity between enhancers and gene promoters [60].

Active enhancers in acinar cells are enriched for binding sites of key acinar lineage-determining transcription factors, notably PTF1A. PTF1A not only binds to promoter regions of digestive enzyme genes but also occupies distal regulatory enhancers that reinforce acinar cell identity [62]. The 134-bp enhancer region of the pancreatic elastase I gene is sufficient to direct pancreatic acinar cell-specific transcription in both transgenic mice and transfected cells in culture [63,64]. Additionally, PTF1A binds to the transcriptional enhancer of the acinar cell-specific *Ela1* gene to activate its transcription, an effect that can be recapitulated in non-pancreatic cell lines [62].

Notably, expression of *Ptf1a* itself is tightly regulated by conserved enhancers. Recent studies have demonstrated that loss-of-function mutations in a single enhancer near *Ptf1a* can lead to pancreatic agenesis and neonatal diabetes [65]. *PTF1A* expression is regulated by at least three distinct regions near the gene [39]. A 14.8-kb region downstream of the last exon is highly conserved across mammals and drives expression in the dorsal spinal cord, but shows little activity in the developing or neonatal pancreas. A nearby 13.4-kb proximal promoter region initiates low-level expression in early acinar progenitors. This promoter activity is strongly enhanced by an adjacent 2.3-kb autoregulatory enhancer, which can robustly drive pancreas-specific expression when tested with a minimal promoter in transgenic mice. In addition, Jiang et al. reported a feed-forward loop formed by the pancreatic transcription factors MIST1 and PTF1A that regulates the differentiated phenotype of adult pancreatic acinar cells [30]. In the adult mouse pancreas, the PTF1 complex drives pancreas-specific transcription through an enhancer located approximately 6 kb upstream of the *Mist1* gene, and the close proximity of PTF1- and MIST1-binding sites further enhance the transcription of target genes in a synergistic manner [30]. These studies highlight a regulatory architecture in which coordinated enhancer usage fine-tunes acinar cell identity and differentiation.

#### Histone modifications

Histone modifications play a central role in regulating gene expression by altering chromatin structure and influencing the accessibility of DNA to transcription factors [66–68]. In general, histone methylation can either activate or repress gene expression, depending on the specific lysine residue modified and the cellular context, whereas histone acetylation is associated with open chromatin state and active transcription [69].

During early pancreatic development, many acinar- and endocrine-specific genes exhibit bivalent chromatin marks, characterized by the simultaneous enrichment of H3K4me3 (trimethylation at lysine 4 of histone H3) and H3K27me3 (trimethylation at lysine 27 of histone H3) [31,70]. Selective loss or retention of these marks correlates with lineage progression toward either a pro-endocrine or pro-acinar cell fate [9,71]. At the DE stage, most (54%) DE signature bivalent genes resolve toward an H3K4me3-dominant active state following removal of H3K27me3, including key transcriptional regulators of endoderm specification exemplified by *GATA6*, which remains enriched for H3K4me3 in acinar cell [32,70]. During subsequent progression to the PE stage, almost all PE signature bivalent genes (87%) are resolved toward H3K4me3 enrichment. These genes include transcription factors central to early pancreatic development, such as *SOX9*, *PDX1*, and *PTF1A* [70]. Similarly, resolution of endocrine regulatory loci toward an H3K4me3-dominant state activates *Ngn3*-centered transcriptional programs that drive endocrine differentiation [72]. In fully differentiated acinar cells, promoters of genes—including those encoding digestive enzymes, components of the secretory machinery, and lineage-defining transcription factors (e.g. *Ptf1a*, *Rbpjl*)—are typically enriched for H3K4me3 [32].

In addition to methylation-based regulation, histone acetylation dynamics also contribute to pancreatic lineage specification. Treatment with histone deacetylase (HDAC) inhibitors alters pancreatic lineage specification by suppressing acinar differentiation while promoting ductal differentiation [73]. Notably, HDAC inhibition enhances endocrine lineage commitment, leading to an expansion of endocrine progenitors and influencing endocrine subtype allocation. Consistently, loss of *Hdac1* during pancreatic development results in defects in exocrine pancreas specification and differentiation in zebrafish [74]. Together, histone modification landscapes not only mark cell states but also regulate transcriptional programs, resolving progenitor plasticity into stable acinar versus endocrine fates.

#### DNA methylation

DNA methylation is a key epigenetic modification involving the covalent addition of a methyl group to the 5-position of cytosine residues within CpG dinucleotides [75]. This modification is catalyzed by DNA methyltransferases (DNMTs): DNMT1 primarily maintains pre-existing methylation patterns, whereas DNMT3A and DNMT3B mediate *de novo* methylation [76,77]. DNA methylation is typically associated with transcriptional repression, as it can hinder the binding of transcription factors and recruit methyl-CpG-binding proteins that promote chromatin compaction [75,78].

Cell type-specific DNA methylation signatures distinguish different pancreatic cell lineages, such as acinar, ductal, and endocrine cells [79]. DNMT1 is essential for the maintenance of highly proliferative acinar cells. Loss-of-function mutations in *Dnmt1* in zebrafish severely compromise the survival of pancreatic acinar cells during development. While endocrine and ductal cell lineages are largely preserved, the majority of acinar cells undergo degeneration by 100 hours post-fertilization [80]. During endocrine differentiation and subsequent maintenance, DNA methylation stabilizes endocrine cell identity by repressing genes associated with alternative fates, thereby preventing lineage switching [81,82]. For example, methylation-dependent repression of the pancreatic α-cell determinant *Arx* restricts activation of α-cell transcriptional networks in β-cells [81]. These findings suggest that DNA methylation supports acinar cell maintenance while reinforcing endocrine cell identity by restricting alternative lineage programs.

Together, histone modification and DNA methylation not only define cell states but actively shape transcriptional programs that resolve progenitor plasticity into stable acinar versus endocrine fates. Importantly, pharmacologic inhibition of histone-modifying enzymes (e.g. HDACs or methyltransferases) provides a powerful and tractable approach to dissect the causal role of chromatin regulation in lineage specification and pancreatic cell fate decisions.

## Post-transcriptional regulation

### RNA splicing

RNA splicing is a tightly regulated enzymatic process in which precursor messenger RNA (pre-mRNA) is processed into mature mRNA within the nucleus [83,84]. Alternative RNA splicing contributes to transcriptomic and proteomic diversity [85,86]. RNA splicing is regulated by splicing-regulatory *cis*-elements within the RNA sequence and *trans*-acting RNA-binding proteins (*trans*-factors) that recognize and interact with these elements [87–89]. Accurate and context-specific RNA splicing is essential for proper development and the maintenance of tissue identity [90]. Mutations in *cis*-elements and/or pathological dysregulation of *trans*-factors can lead to aberrant splicing, which is frequently implicated in tumorigenesis. Consequently, the targeted modulation of abnormal splicing events represents a promising therapeutic strategy [91,92].

Alternative RNA splicing is tightly regulated in pancreatic acinar cells to maintain cellular homeostasis. Downregulation of the splicing factor SRSF1 serves as a negative feedback mechanism in response to oncogenic KRAS^G12D^ signaling [33]. In addition to canonical spliceosome-mediated splicing in the nucleus, acinar cells also rely on a distinct, stress-responsive noncanonical splicing mechanism to preserve cellular function. X-box-binding protein 1 (XBP1) plays a critical role in maintaining endoplasmic reticulum (ER) homeostasis in pancreatic acinar cells [93]. Unlike typical pre-mRNA splicing, which occurs in the nucleus via the spliceosome, *XBP1* mRNA is spliced in the cytoplasm by the endoribonuclease IRE1α, an ER membrane–resident protein [94]. Upon ER stress, IRE1α becomes activated, oligomerizes, and cleaves a specific 26-nucleotide intron from cytoplasmic unspliced *XBP1* (*XBP1u*) mRNA. The two resulting exons are then ligated by the tRNA ligase RTCB. This splicing creates a frame-shift, producing the *XBP1s* (spliced) isoform, which encodes an active transcription factor that enters the nucleus to upregulate unfolded protein response (UPR) target genes. In acinar cells, *XBP1s* is essential for supporting secretory function and preventing ER stress–induced apoptosis [93,95]. Loss of XBP1 impairs acinar differentiation, reduces digestive enzyme production, and leads to disorganization of the secretory machinery [96].

### Protein translation and ER protein folding

Pancreatic acinar cells sustain exceptionally high rates of protein translation to support digestive enzyme production [97]. These cells contain an extensive RER, which functions as the primary site of secretory protein synthesis, folding, and entry into the secretory pathway [98]. Secretory proteins carry N-terminal signal sequences recognized by the signal recognition particle, which delivers the ribosome–nascent chain complex to the ER membrane and transfers it to the Sec61 translocon for translocation into the ER lumen. Once inside the ER, proteins undergo chaperone-assisted folding and covalent modifications, including N-linked glycosylation and disulfide bond formation [99]. They are then transported to the Golgi apparatus, where they are further modified, sorted, and packaged into zymogen granules that accumulate at the apical pole of the acinar cell [100]. Protein synthesis in acinar cells is tightly regulated by hormonal and neurocrine signals. Stimulation by factors such as cholecystokinin, a key gastrointestinal hormone, insulin, and cholinergic agonists occurs rapidly, primarily through translational regulation, and is notably dose-dependent, combinatorial, and protein-specific [101–104].

The intense biosynthesis in acinar cells places a substantial burden on the secretory pathway and requires tight coordination between nascent protein synthesis and ER folding capacity to maintain ER proteostasis. Disruption of ER homeostasis activates maladaptive UPR pathways and promotes pancreatic pathology. For example, loss of the ER acetyl-CoA transporter AT-1 induces chronic ER stress in acinar cells, activates XBP1 and protein kinase R-like ER kinase signaling, and leads to pancreatitis characterized by intracellular trypsin accumulation, inflammation, and fibrosis [105]. Another example linking protein-folding burden to pancreatic pathology is provided by PRSS1 overaccumulation, which induces ER stress in acinar cells [106]. Consistently, elevated expression of ER stress markers, including ATF6, XBP1, and CHOP, has been observed in both human chronic pancreatitis samples and PRSS1 transgenic mouse models. These observations suggest that tight coordination between high-rate protein translation and ER folding capacity is essential to maintain proteostasis in acinar cells.

### MicroRNAs

miRNAs are ∼22-nucleotide, non-coding single-stranded RNAs that bind predominantly to the untranslated regions of target mRNAs, leading to translational repression or mRNA degradation [107,108]. miRNAs are predicted to regulate more than 60% of protein-coding genes and are key regulators of diverse cellular processes, including growth, differentiation, proliferation, and apoptosis [109–113]. miRNAs contribute to lineage specification and play important roles in pancreatic development. Let-7g, Let-7a, miR-200a, miR-127, and miR-375 are endocrine-enriched miRNAs that promote endocrine differentiation by targeting transcription factors involved in cell cycle regulation [114]. In the exocrine lineage, miR-18a fine-tunes the regulation of pancreatic progenitors and exocrine cells by repressing *Ptf1a* expression [115]. These findings suggest that miRNAs regulate pancreatic development and maintenance by fine-tuning transcriptional networks and reinforcing cell identity. Defining the underlying regulatory mechanisms and direct miRNA targets will be critical to fully elucidate how miRNAs regulate pancreatic development and acinar cell homeostasis.

## Transcriptome dysregulation as a driver of acinar cell plasticity and ADM

**Figure 3:**
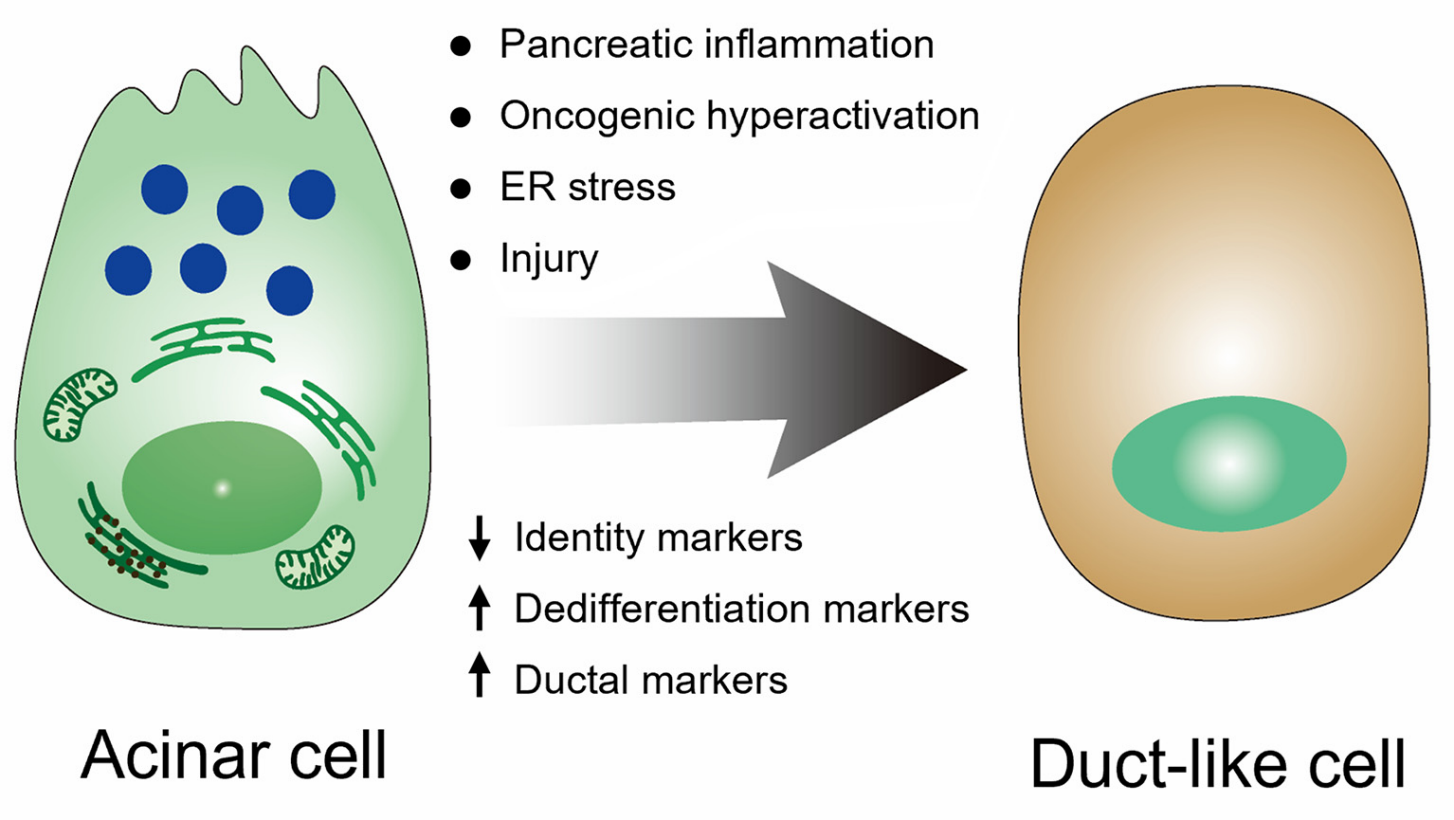
Acinar-to-ductal metaplasia. Pancreatic injury, oncogenic activation, and inflammatory signaling drive acinar cell dedifferentiation and transdifferentiation into a duct-like phenotype that supports tissue repair. Under sustained oncogenic and growth factor signaling, this ADM becomes irreversible and progresses to precancerous lesions such as PanIN.

Among adult pancreatic cell lineages, acinar cells exhibit the highest degree of plasticity [116,117]. Acinar cells undergo ADM in response to diverse stress signals, including inflammation, tissue injury, unresolved ER stress, and oncogenic KRAS activation [7,10,118]. This process is characterized by loss of polarity and secretory architecture, reduced expression of digestive enzymes, and activation of ductal gene programs (Figure 3) [119–121]. The role of ADM in pancreatic tumorigenesis was initially demonstrated in mice with TGF-α overexpression, which induced acinar cell dedifferentiation and pancreatic metaplasia, and later recapitulated in 3D culture systems, in which EGFR activation by TGF-α or other growth factors efficiently induces ADM in mouse acinar cells [119,122,123]. In the context of pancreatic regeneration, transient ADM represents an adaptive, reversible reprogramming process that facilitates tissue repair. By downregulating the secretory program, acinar cells reduce cellular stress associated with enzyme production and enhance their capacity to survive injury, proliferate, and subsequently redifferentiate into functional acinar cells [10].

During acute pancreatitis (AP), ADM serves as a protective program that minimizes autodigestive damage [7]. However, in chronic pancreatitis, repeated injury leads to persistent ADM, which may become irreversible in the presence of oncogenic KRAS mutations and chronic inflammation [124,125]. This pathological stabilization of the duct-like state creates a permissive environment for the development of precursor lesions, most notably PanIN, which can accumulate additional genetic and epigenetic alterations that drive malignant transformation [10,11]. Consequently, ADM represents a central biological intersection point linking pancreatic injury, inflammation, and cancer initiation [120]. Understanding the molecular and epigenetic mechanisms that govern the reversibility or irreversibility of ADM is critical for developing strategies to promote pancreatic regeneration while preventing neoplastic progression.

### Transcriptional dysregulation in ADM

The loss of lineage-defining transcription factors is a hallmark of transcriptomic reprogramming during ADM [118]. Expression of PTF1A, GATA6, and MIST1, which are essential for acinar cell differentiation and maintenance of secretory function, is markedly downregulated, while the activity of ductal and progenitor-associated transcription factors such as SOX9, HNF1B, SOX11, and SOX4 is increased, leading to the activation of ductal and progenitor-like transcriptional programs [45,118,126]. For example, induction of SOX9 is an early and essential event in KRAS-driven pancreatic tumor initiation [127]. It promotes a ductal transcriptional program while facilitating the loss of acinar identity during ADM. In addition, Krüppel-like factor 4, a zinc-finger transcription factor involved in epithelial differentiation and cellular reprogramming, promotes ADM by suppressing acinar identity and activating ductal gene expression [128].

Transient silencing of MIST1 during AP is a critical event that allows acinar cells to survive AP injury by suppressing their secretory function and permitting a window of cell proliferation. However, to fully re-establish a functional acinar cell capable of efficient exocytosis, the *Mist1* gene must be reactivated to scale up the appropriate intracellular machinery that generates secretory vesicles, expands the ER, and establishes cell communication via gap junction signaling [129]. Consistently, reactivation of acinar transcriptional networks can reverse dedifferentiation and suppress ADM-associated phenotypes. Re-expression of *Ptf1a* in established PanINs reverts them to quiescent acinar cells *in vivo* [130]. Similarly, induced expression of MIST1 or PTF1A rescues the acinar gene expression and decreases tumorigenic properties in pancreatic cancer cells, and tamoxifen-inducible expression of E47 reprograms human tumor cells toward a quiescent acinar state [131,132].

### Role of histone modifications and DNA methylation in lineage reprogramming

Changes in histone modifications, particularly the loss of H3K27ac and gain of repressive H3K27me3 marks at acinar-specific loci, contribute to silencing of the acinar gene program during ADM [32,133,134]. Additionally, abnormal DNA methylation patterns can impair the expression of critical transcription factors and digestive enzymes. For instance, DNMT1 is indispensable for acinar cell survival and homeostasis, and its depletion leads to acinar cell degeneration [80,135]. A recent study by Lo et al. found that DNA methylation changes during the ADM transition establishes an epigenetic memory that promotes cancer-associated cellular plasticity, even in the absence of oncogenic mutations [136]. These findings indicate that epigenetic remodeling reinforces acinar gene silencing and sustains cellular plasticity during ADM.

### Post-transcriptional regulation of ADM

RNA splicing plays a central role in regulating acinar cell plasticity. Recent studies have identified the splicing factor SRSF1 as a key determinant of ADM. Under homeostatic conditions, SRSF1 levels are tightly controlled in acinar cells; however, its aberrant upregulation is sufficient to induce ADM and recapitulate features of pancreatitis [33]. Conversely, depletion of SRSF1 attenuates MAPK signaling and suppresses ADM induction [33]. Additionally, unconventional splicing of *XBP1* mRNA provides an additional layer of post-transcriptional regulation. The active isoform *XBP1s* supports the high secretory load of acinar cells and prevents ER stress–induced apoptosis [96].

Notably, several disease-associated variants in pancreatitis-related genes disrupt pre-mRNA splicing. Chymotrypsin C (*CTRC*) encodes a digestive protease secreted by pancreatic acinar cells that regulates intrapancreatic trypsin activity and protects against premature trypsin activation [137,138]. Beer et al. identified the synonymous *CTRC* c.132G>A (p.Q44 =) variant as a functionally deleterious mutation that induces aberrant pre-mRNA splicing and reduces *CTRC* expression [139,140]. In addition, *SPINK1* (serine protease inhibitor Kazal type 1) encodes a key endogenous inhibitor of trypsin that protects the pancreas from autodigestion [141]. The *SPINK1* c.194G>A (p.R65Q) variant alters the last nucleotide of exon 3, disrupts the exon 3 splice donor site, and results in defective pre-mRNA splicing with markedly reduced mRNA and protein expression [140]. These findings highlight aberrant splicing as a pathogenic risk factor in pancreatitis and suggest that therapeutic strategies modulating RNA splicing may offer a promising approach to restore correct transcript processing and gene function [142–144].

miRNA networks constitute an additional layer of post-transcriptional regulation that cooperates with transcriptional and epigenetic programs to reshape acinar cell identity [145,146]. Loss of acinar-enriched miR-216a, miR-216b, or miR-217 promotes acinar cell dedifferentiation, enhances ADM, and delays recovery after pancreatic injury [35]. In addition, Ge et al. reported that reduced levels of miR-802, a regulator of normal pancreatic acinar function, promotes ADM and acinar cell proliferation, ultimately triggering AP and aggravating pancreatic injury, whereas restoring miR-802 expression suppresses ADM and mitigates disease progression [147]. Similarly, Let-7b and miR-495 repress HNF6, a transcription factor whose aberrant upregulation during ADM drives ductal gene expression and cellular reprogramming [148]. Broad impairment of miRNA biogenesis—for example, through DICER deletion—induces profound acinar cell plasticity and spontaneous ADM, underscoring the centrality of miRNA-dependent pathways in maintaining acinar identity [10,145,149].

### ER stress in ADM

Disruption of ER proteostasis activates UPR signaling pathways that promote acinar cell plasticity and facilitate ADM. For example, loss of the ER-resident protein VMP1 impairs autophagy, leading to the accumulation of SQSTM1/p62, activation of NRF2 signaling, and exacerbation of ER stress, thereby promoting ADM, inflammation, and fibrosis [150]. Consistently, the UPR effector activating transcription factor 3 (ATF3) is rapidly induced during pancreatic injury and occupies regulatory regions of genes involved in inflammation, acinar differentiation, and cell–cell junctions. Genome-wide analyses show that ATF3 is enriched at regulatory regions of more than 30% of differentially expressed genes during the initiation of pancreatitis. Mechanistically, ATF3 promotes recruitment of HDACs to the promoter of *Mist1*, leading to transcriptional repression [151]. These findings establish that transcriptional reprogramming induced by ER stress plays a central role in shaping acinar cell fate.

## Conclusion

Pancreatic acinar cell identity is sustained by a highly coordinated, multilayered regulatory architecture that integrates transcriptional, epigenetic, and post-transcriptional mechanisms [13,16,33,38,62,70,115,118]. Core transcription factors establish acinar-specific gene expression, which are further reinforced by enhancer activity, chromatin accessibility, histone modifications, and DNA methylation. Superimposed on this framework, post-transcriptional regulation—such as alternative splicing, translational control, and miRNA-mediated mechanisms—provides dynamic tuning of gene expression, enabling acinar cells to sustain their extraordinary secretory capacity while retaining adaptive flexibility [94,105,129]. These regulatory mechanisms are functionally interconnected. Perturbations such as inflammatory injury, ER stress, or oncogenic KRAS signaling disrupt this integrated network, leading to widespread transcriptomic reprogramming and ADM [35,45,118,124,126,150]. A central unresolved question is what defines the transcriptome threshold between acinar cell homeostasis and ADM.

Looking forward, a major priority will be to define the key transcriptomic checkpoints that control acinar cell state transitions and to elucidate how signaling pathways interface with RNA regulatory mechanisms to enforce or destabilize cell identity. High-resolution approaches, including single-cell and long-read transcriptomics, combined with functional perturbation of RNA regulatory factors, will be essential to elucidate these networks with mechanistic precision [152–154]. In parallel, pharmacological interventions—such as epigenetic modulators and antisense oligonucleotides that directly modulate splicing events—offer powerful tools for interrogating the functional relevance of specific regulatory mechanisms [155,156]. Importantly, these strategies also hold promise as therapeutic approaches to restore acinar identity, constrain pathological transformation, and intercept early events in pancreatic tumorigenesis.

## Perspectives

- Pancreatic acinar cell plasticity and ADM represent a central biological intersection linking pancreatic homeostasis, adaptive injury responses, and early events in tumor initiation, thereby serving as a critical framework for understanding the mechanisms underlying pancreatitis development and the progression to pancreatic cancer.
- Current evidence suggests that coordinated transcriptional, epigenetic, and post-transcriptional mechanisms regulate acinar cell identity, the reversibility of ADM, and its progression toward pathological reprogramming, particularly in response to cellular stress and oncogenic signaling.
- Recent advances in primary acinar cell culture, acinar-derived organoids, and three-dimensional co-culture systems provide a powerful means to dissect molecular mechanisms, validate candidate regulators, and test therapeutic strategies, complementing *in vivo* studies to achieve a more comprehensive understanding of acinar cell transcriptional programs in health and disease. Future work should define precise transcriptomic checkpoints and exploit emerging RNA- and epigenetic-based therapies to restore homeostasis, reverse ADM, and block early neoplastic transformation.

## Data Availability

Data sharing is not applicable to this article as no new datasets were generated or analyzed.

## Open Access

Open access for this article is provided via our Subscribe to Open programme, made possible through the support of our subscribing institutions. Learn more here: https://portlandpress.com/pages/open_access_options_and_prices#conditional.

## Abbreviations

ADM: acinar-to-ductal metaplasia
AP: acute pancreatitis
ATF3: activating transcription factor 3
bHLH: basic helix–loop–helix
CTRC: chymotrypsin C
DNMT: DNA methyltransferase
ER: endoplasmic reticulum
GATA: guanine–adenine–thymine–adenine zinc finger transcription factors
HDAC: histone deacetylase
miRNA: microRNA
MPC: multipotent progenitor cell
PanIN: pancreatic intraepithelial neoplasia
PDAC: pancreatic ductal adenocarcinoma
PDX1: pancreatic and duodenal homeobox 1
PE: pancreatic endoderm
PTF1A: pancreas transcription factor 1 subunit alpha
RER: rough endoplasmic reticulum
UPR: unfolded protein response
XBP1: X-box-binding protein 1
KRAS: Kirsten rat sarcoma virus oncogene homologue
TGF-α: Transforming growth factor alpha
